# Plutonium concentration and isotopic ratio in soil samples from central-eastern Japan collected around the 1970s

**DOI:** 10.1038/srep09636

**Published:** 2015-04-16

**Authors:** Guosheng Yang, Jian Zheng, Keiko Tagami, Shigeo Uchida

**Affiliations:** 1 Research Center for Radiation Protection, National Institute of Radiological Sciences, Anagawa 4-9-1, Inage, Chiba 263-8555, Japan

## Abstract

Obtaining Pu background data in the environment is essential for contamination source identification and assessment of environmental impact of Pu released from the Fukushima Daiichi nuclear power plant (FDNPP) accident. However, no baseline information on Pu isotopes in Fukushima Prefecture has been reported. Here we analyzed 80 surface soil samples collected from the central-eastern Japan during 1969–1977 for ^239+240^Pu activity concentration and ^240^Pu/^239^Pu atom ratio to establish the baseline before the FDNPP accident. We found that ^239+240^Pu activity concentrations ranged from 0.004 –1.46 mBq g^−1^, and ^240^Pu/^239^Pu atom ratios varied narrowly from 0.148 to 0.229 with a mean of 0.186 ± 0.015. We also reconstructed the surface deposition density of ^241^Pu using the ^241^Pu/^239^Pu atom ratio in the Japanese fallout reference material. The obtained results indicated that, for the FDNPP-accident released ^241^Pu, a similar radiation impact can be estimated as was seen for the global fallout deposited ^241^Pu in the last decades.

The Fukushima Daiichi Nuclear Power Plant (FDNPP) accident in 2011 resulted in trace release of the reactor core Pu into the atmosphere after intentional venting operation and reactor hydrogen explosions^1^,^2^,^3^,^4^,^5^,^6^,^7^. Atmospheric Pu was subsequently deposited on the ground by the wet and dry deposition processes^8^. To estimate the environmental impact of the FDNPP source Pu, background data on Pu distributions in the environment before the FDNPP accident are critical.

Due to the difficulty of Pu analysis, baseline information on Pu activity in Japanese soils is very limited. Yamamoto et al.^9^ examined the concentrations of ^239+240^Pu for 30 rice-field surface soil samples collected from 15 locations in Japan mostly in 1963 and 1976, and for 15 soil samples periodically collected from 2 locations during 1957-1980. Their study found the integrated deposits of Pu isotopes on the Japan Sea coast of Honshu were 2.5 to 3 times higher than those on the Pacific coast, and the concentrations of ^239+240^Pu in rice-field soils ranged from 0.078 to 1.43 mBq g^−1^. A similar ^239+240^Pu concentration range of 0.07–0.7 mBq g^−1^ was observed in agricultural upland fields soils in Rokkasho, Aomori Prefecture^10^. In addition, ^239+240^Pu concentration in surface layer (0–5 cm) soil samples collected in 1995 in Kyushu was reported to range from 0.50–0.65 mBq g^−1^ 11. A more recent study conducted by Muramatsu et al.^12^ analyzed ^239+240^Pu concentrations in 20 soil samples collected from agricultural fields (vegetable/wheat fields and rice paddy fields) and forests in several places in Japan. They found the range of the ^239+240^Pu concentrations to be 0.15–4.31 mBq g^−1^; the highest concentration of 4.31 mBq g^−1^ was found in a surface soil (0–2 cm) sample collected from a forest in Aomori Prefecture. In addition to the Pu activity, the Pu isotopic ratio is an important fingerprint for contamination source identification. The ^240^Pu/^239^Pu atom ratio is of special interest; for nuclear tests this ratio changes with the weapon type and yield, while for nuclear reactors it changes with the reactor type and nuclear fuel burn up. Therefore, the ^240^Pu/^239^Pu atom ratio can provide valuable information on the nature of the Pu emitting source^13^,^14^. In previous investigations to obtain the background data for Pu in Japanese soil, the ^240^Pu/^239^Pu atom ratio was found to be in the range of 0.14–0.24, which revealed that the major source of Pu in the environment was global fallout from the atmospheric nuclear explosions conducted in the last century^9^,^10^,^11^,^12^,^13^,^14^,^15^.

However, to our knowledge, no baseline information on Pu activity distribution and atom ratio in Fukushima Prefecture are available for times prior to the 2011 nuclear accident. From 1967 to1994, the National Institute of Radiological Sciences (NIRS) collected surface soils mainly from school grounds in Japan to establish a surface soil database for understanding the natural radiation level. Therefore, in this work, we used the NIRS archived soil samples collected in during 1969 to 1977 from Fukushima and its adjacent Prefectures in central-eastern Japan (Fig. 1) to establish background data on the ^239+240^Pu activity concentrations and ^240^Pu/^239^Pu atom ratio. In addition, since a high ^241^Pu/^239+240^Pu activity ratio (higher than 100), was observed in the FDNPP-source Pu^1^, we also reconstructed ^241^Pu activities in the analyzed soil samples based on the ^241^Pu/^239^Pu atom ratio in the Japanese fallout reference material. The obtained background data are important to estimate the radiation dose due to the deposition of global fallout Pu and the FDNPP-source Pu.

## Results

The results of activity concentrations of ^239+240^Pu and the atom ratios of ^240^Pu/^239^Pu in all soil samples are summarized in Table S1 in the Supplementary Material, and the mean values of activities of ^239+240^Pu and the atom ratios of ^240^Pu/^239^Pu in each Prefecture are presented in Table 1. The ^239+240^Pu activities in school ground soils are quite low, ranging from 0.004 to 0.412 mBq g^−1^. In the two samples collected from residential area in Okuma and Futaba, Fukushima Prefecture, ^239+240^Pu activities were 0.294 and 0.695 mBq g^−1^, respectively. The highest ^239+240^Pu activity of 1.46 mBq g^−1^ in this study was found in the soil collected from the grounds of one Park in Sendai, Miyagi Prefecture. Fig. 2a shows the frequency distribution of ^239+240^Pu activities in soil samples (school grounds, residential areas and park grounds). Among the 80 soil samples analyzed, 48.8% were lower than 0.05 mBq g^−1^, and 77.5% were lower than 0.15 mBq g^−1^.

The ^240^Pu/^239^Pu atom ratios of all samples are plotted against ^239+240^Pu activity concentrations in Fig. 3. It can be seen that the ^240^Pu/^239^Pu atom ratios ranged from 0.148 to 0.229 in the investigated school grounds soil. For the two samples from the residential areas and the one sample from the park grounds, the ^240^Pu/^239^Pu atom ratios ranged from 0.182 to 0.188. The frequency distribution of ^240^Pu/^239^Pu atom ratios in all samples is plotted in Fig. 2b. A typical Gaussian distribution was obtained. Among the 80 soil samples, 30 samples had ^240^Pu/^239^Pu atom ratios ranging from 0.18–0.19, and 58 samples had ^240^Pu/^239^Pu atom ratios of 0.17–0.20. The mean ^240^Pu/^239^Pu atom ratio was 0.186 ± 0.015, which is similar to that of global fallout (0.180 ± 0.007)^13^, indicating that global fallout Pu deposition was the major source, although a small contribution of the Chinese Nuclear Tests at Lop Nor has been observed in fallout samples collected in the 1970s in Japan^16^.

## Discussion

Eighty soil samples in this study were collected from central-eastern Japan in the period from 1969 to 1977. From 1945 to 1980, 543 atmospheric nuclear tests were conducted worldwide, and during 1964–1980, 22 atmospheric nuclear weapons tests were conducted by China at Lop Nor^17^. The Nagasaki atomic bomb detonation on August 9, 1945 released Pu into the environment; a study on the geographic distribution of Nagasaki atomic bomb-derived Pu indicated that plutonium from the atomic bomb was deposited in the eastern area from the hypocenter reaching up to 100 km eastwards^18^. In addition, there was no detectable deposition of Chernobyl accident-sourced Pu in 1986, so Pu in the soil samples of the present study was assumed to have come mainly from the global fallout due to the atmospheric nuclear tests. The ^239+240^Pu activity concentrations in our study were in the range of 0.004–1.46 mBq g^−1^, which were comparable with previous studies on the activity levels of Pu in soils in Japan before the FDNPP accident (0.07–4.31 mBq g^−1^) as shown in Table 2. Xu et al.^19^ demonstrated that the combined effects of many environmental factors were responsible for the variation of Pu concentrations in the surface soils. We compared the activity concentrations of ^239+240^Pu with organic contents in the investigated surface soil samples; however, no significant correlation between Pu activities and organic matter contents was found. Therefore, more detailed information should be obtained in the future to reveal the variation of concentrations in the surface soils.

As shown in Table 1, higher ^239+240^Pu activity concentrations can be seen in the four prefectures of Iwate, Ibaraki, Miyagi, and Fukushima, with mean values of 0.154 ± 0.131, 0.134 ± 0.075, 0.103 ± 0.101, and 0.091 ± 0.085 mBq g^−1^, respectively. While for Tokyo and the four prefectures, Tochigi, Gunma, Saitama and Chiba, significantly lower mean values varying narrowly from 0.038–0.055 mBq g^−1^ were observed. The similar variation trend was seen in the surface deposition density (0–5 cm) of ^239+240^Pu as shown in Fig. 4. The surface deposition density of ^239+240^Pu ranged from 0.3 Bq m^−2^ to 27 Bq m^−2^ for the school grounds soil samples. Relatively higher ^239+240^Pu surface inventories of 19–45 Bq m^−2^ were found for two residential surface soil samples, and of 95 Bq m^−2^ for surface soil sample of park grounds. Recently, a large scale investigation on the surface deposition of Pu in soils collected at 100 sites in September 2013 northwest of the FDNPP was reported^20^. It was found that the surface deposition density (Bq m^−2^) ranged from 0.35 to 40; these values are comparable to those we detected in the soils collected in the 1970s, indicating that the FDNPP accident did not cause significant increase of Pu deposition in central-eastern Japan.

However, alteration of the ^240^Pu/^239^Pu atom ratio in various environmental samples was significant after the FDNPP accident. Fig. 5 compares the atom ratios of ^240^Pu/^239^Pu in different environmental samples contaminated by the FDNPP accident and the background data of ^240^Pu/^239^Pu atom ratios in environmental samples collected before the FDNPP accident. Regarding the background ^240^Pu/^239^Pu atom ratio in the Japanese environment, in this investigation, we found that the fingerprint of Pu (^240^Pu/^239^Pu atom ratios) in soil from central-eastern Japan varied narrowly from 0.148 to 0.229 (with a mean of 0.186 ± 0.015) as mentioned above. Kelley et al.^13^ reported ^240^Pu/^239^Pu atom ratios in two surface soil samples from Tokyo and Sapporo (in northern Japan) were 0.1755 ± 0.0012 and 0.1765 ± 0.0011, which fall into the interval in our study. During the period after the Chernobyl accident in 1986 and before the Fukushima accident in 2011, ^240^Pu/^239^Pu atom ratios in Japanese soils were in the range of 0.14–0.24^10^,^11^,^12^. We can conclude, therefore, that the release of Pu from the Chernobyl accident resulted in almost no change in the Pu contamination in Japanese soil. After the FDNPP accident, obviously high atom ratios of ^240^Pu/^239^Pu were found in several environmental samples. Zheng et al.^1^ and Yamamoto et al.^5^ noted that atom ratios of ^240^Pu/^239^Pu in litter samples and black substances (road dust) collected in northwest of the FDNPP site varied very narrowly from 0.323 to 0.335, and from 0.285 to 0.365, respectively. Zheng et al.^1^ also measured the atom ratio of ^240^Pu/^239^Pu in one surface soil sample (0–2 cm) collected 20 km south of the FDNPP site and obtained the value of 0.303 ± 0.030. Schneider et al.^3^ reported a high ^240^Pu/^239^Pu atom ratio in one vegetation sample (0.381 ± 0.046). Recently, Shinonaga et al.^6^ demonstrated that the FDNPP accident-released Pu was transported 120 km after the FDNPP accident by measuring the Pu atom ratio in aerosol samples (they obtained atom ratios of ^240^Pu/^239^Pu of 0.333–0.426). Nishihara et al.^21^ have estimated the possible atom ratios of ^240^Pu/^239^Pu that existed in March 2011 in the FDNPP reactor cores (0.320–0.356) and spent fuel pools (0.394–0.468) using the ORIGEN2 code and the fuel burn-up data from the Tokyo Electric Power Company. These comparisons have clearly indicated the wide distribution of the FDNPP accident-released Pu in the environment.

Among the Pu isotopes released from severe reactor accident, like the Fukushima nuclear accident, ^241^Pu is important for radiation dose estimation. It is a beta-emitter with a half-life of 14.4 years. With its decay, the ingrowth of ^241^Am (alpha and gamma-emitter, T_1/2_ = 432.7 years) will present a new radiation risk^1^. The release of ^241^Pu with a high ^241^Pu/^239+240^Pu activity ratio (> 100) from the FDNPP accident was first reported by Zheng et al.^1^ by the analysis of litter samples in Fukushima Prefecture. Recently, Ikeuchi^20^ reported a comprehensive investigation on the deposition of the FDNPP accident-released Pu isotopes in surface soils in Fukushima Prefecture, and confirmed the wide distribution of FDNPP-sourced ^241^Pu. To better understand the radiation impact of the FDNPP-sourced ^241^Pu in the environmental, the background data on the deposition level of ^241^Pu, especially in the period of the peak of global fallout is critical.

Due to the low activities of ^241^Pu in the school ground soils after five decades of decay, ^241^Pu was below the detection limit of the analytical method (1 mBq/g), and thus not detected in this study. In order to establish background data of the ^241^Pu activity in the analyzed soil samples, we used the ^241^Pu/^239^Pu atom ratio of 0.00261 ± 0.00026 (^241^Pu decay reference to January 1, 2000) in fallout reference material reported by Zhang et al.^16^. This reference fallout material was prepared from fallout deposition samples collected monthly at 14 stations throughout Japan in 1963–1979^22^. Since the fallout reference material and the soils samples we investigated in this study were sampled in the period after large scale atmospheric nuclear weapons tests and before the widespread operation of nuclear power plants in Japan, we consider that they have the same source of Pu isotopes, *i.e.* mainly from the global fallout with a small contribution from the Chinese nuclear tests, therefore, we can use the ^241^Pu/^239^Pu atom ratio detected in the fallout reference material to re-construct the ^241^Pu activities in the soil samples we analyzed. To understand the highest background level of ^241^Pu activity in the soil samples, decay of ^241^Pu was corrected to January 1, 1964, the year of peak deposition of global fallout Pu^17^. As shown in Fig. 6, the re-constructed ^241^Pu activities were highly correlated with the ^239+240^Pu activities, thus the ^241^Pu/^239+240^Pu activity ratio of 14.8 was obtained. Since the half-lives of ^239^Pu and ^240^Pu are very long (2.411 × 10^4^ y and 6.563 × 10^3^ y, respectively), in the timescale of several decades, the ^239+240^Pu activity can be considered unchanged, the ^241^Pu/^239+240^Pu activity ratio of 14.8 obtained in this study can be used to estimate ^241^Pu background in soils collected in other areas in Japan, once ^239+240^Pu activity is measured. The estimated ^241^Pu/^239+240^Pu activity ratio in the present study is slightly higher than that of global fallout (ca. 12.1) obtained from lake sediments^23^.

The estimated ^241^Pu activities are summarized in Table S1. It was found that the ^241^Pu activities ranged from 0.06 to 6.07 mBq g^−1^ in 77 school grounds soil samples. Higher ^241^Pu activities were found in the soil samples collected from residential areas and the park grounds, ranging from 4.28 mBq g^−1^ to 21.25 mBq g^−1^. It was noted that the distribution patterns of measured ^239+240^Pu activities and the estimated ^241^Pu activities were quite similar. For the ^241^Pu activities, among the investigated soil samples, 89.6% were less than 0.3 mBq g^−1^. The northern prefectures of Ibaraki, Fukushima, Miyagi and Iwate presented relatively higher ^241^Pu activities than those in the four southern prefectures (Tochigi, Gunma, Saitama, and Chiba) and Tokyo.

Fig. 7 showed the re-constructed surface deposition density of ^241^Pu (Bq m^−2^) in central-eastern Japan in the period of the peak global fallout in 1964. The surface deposition density of ^241^Pu ranged from 3.9 to 394 Bq m^−2^. Similar to the distribution of ^239+240^Pu, higher deposition was seen in the four northern prefectures. The recent study reported by Ikeuchi^20^ indicated that the FDNPP-sourced ^241^Pu was detected in 41 sites among the 100 sites investigated in Fukushima Prefecture. The surface deposition density (0–5 cm) of ^241^Pu varied from 12 to 240 Bq m^−2^; this is in the same levels as the ^241^Pu deposition in 1964. Therefore, we consider that although the release and deposition of ^241^Pu with extremely high ^241^Pu/^239+240^Pu activity ratios (0.96–129.6) was widely detected in the environment after the FDNPP accident, a similar radiation impact can be estimated as that of the global fallout deposited ^241^Pu during the last five decades.

## Methods

### Reagents and materials

High-purity water (18 MΩ cm^−1^) was prepared with a Millipore Milli-Q-Plus water purification system. All chemicals (HCl, HNO_3_, NaNO_2_, NH_4_I, H_2_O_2_, HBr) were of analytical grade, except for the final solution preparation for the ICP-MS measurement, in which ultrapure grade 68% HNO_3_ (Tama Chemicals, Japan) was used. The two anion-exchange resins, AG 1X8 (100–200 mesh, Cl-form) and AG MP-1M (100–200 mesh, Cl-form) were obtained from Bio-Rad, which were packed in a Muromac mini-column (M type, 6.5–8.5 mm × 58 mm i.d.) for Pu separation and purification.

^242^Pu (CRM 130, plutonium spike assay and isotopic standard, New Brunswick Laboratory, USA) was used to spike the soil samples as a yield tracer. The mixed Pu isotope standard solution (NBS-947) with certified ^240^Pu/^239^Pu atom ratio of 0.242 was employed for mass bias correction. Two soil standard reference materials (IAEA-soil-6 and IAEA-375) were used to validate our analytical method.

**Figure 1:**
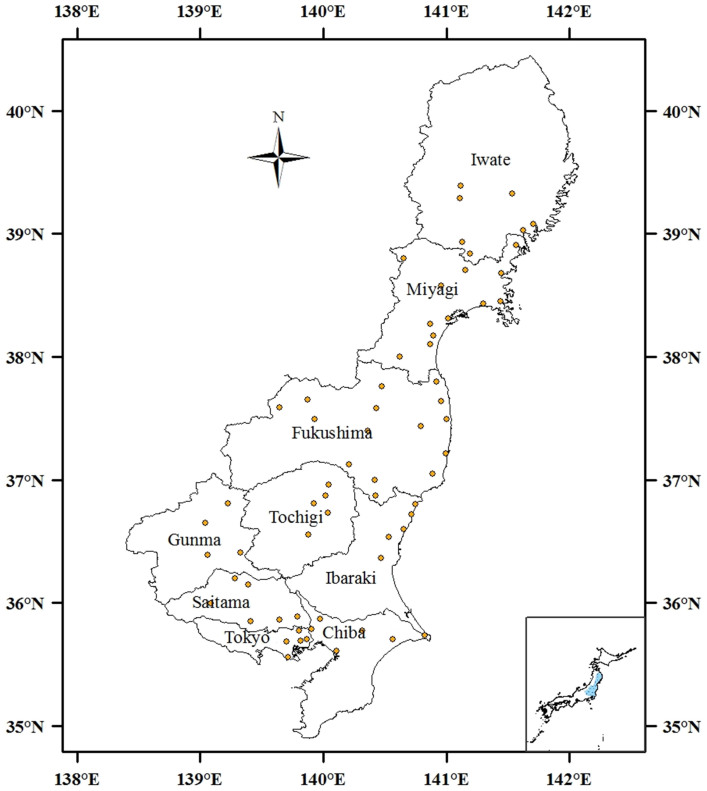
Map showing the soil sampling locations in central-eastern Japan. This map was prepared with Arc GIS 10.0 software.

**Figure 2:**
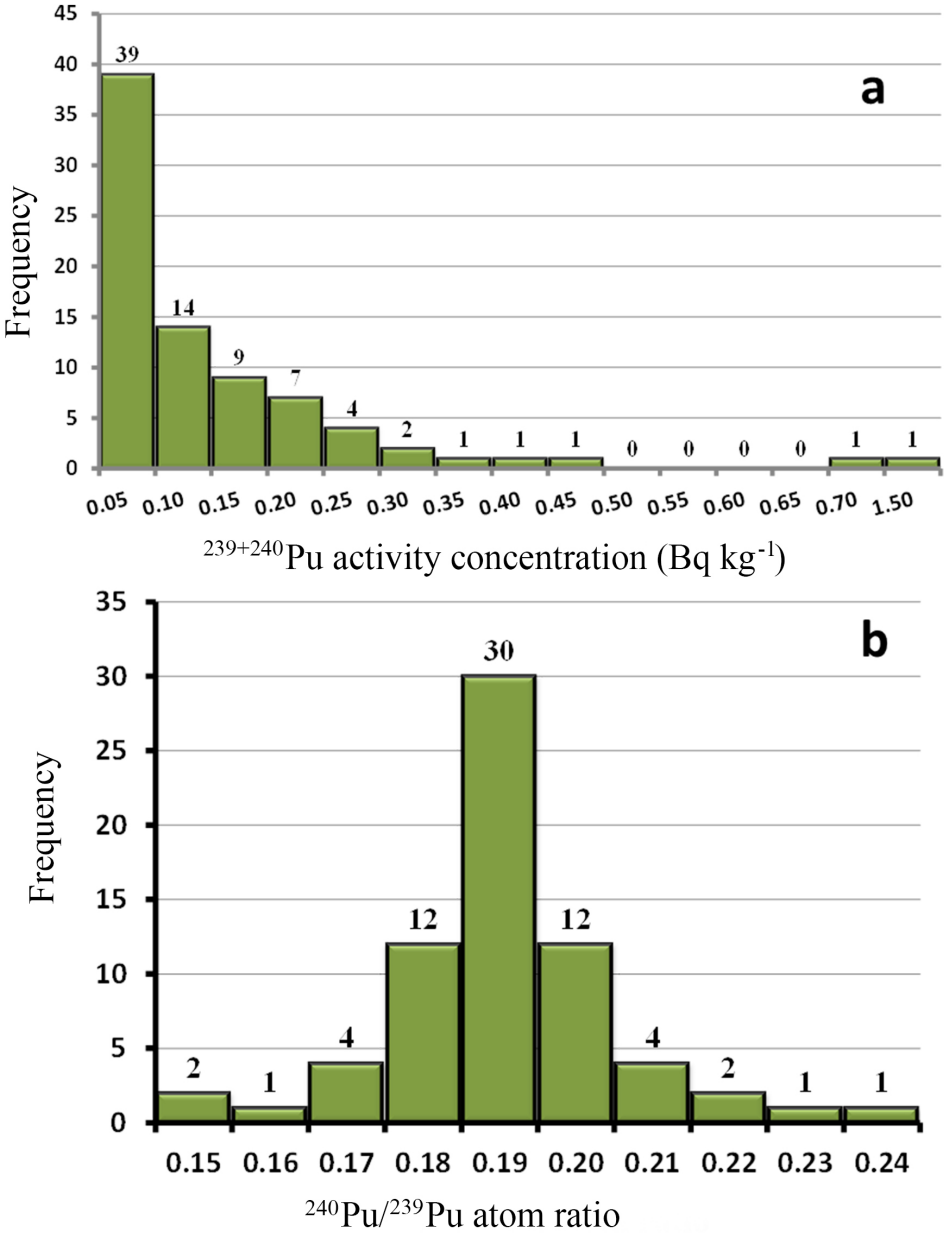
Frequency distributions of ^239+240^Pu activity concentrations (mBq g^−1^) (a), and ^240^Pu/^239^Pu atom ratios (b) in soil samples (school ground, residential areas and park grounds) collected in central-eastern Japan in the 1970s.

**Figure 3:**
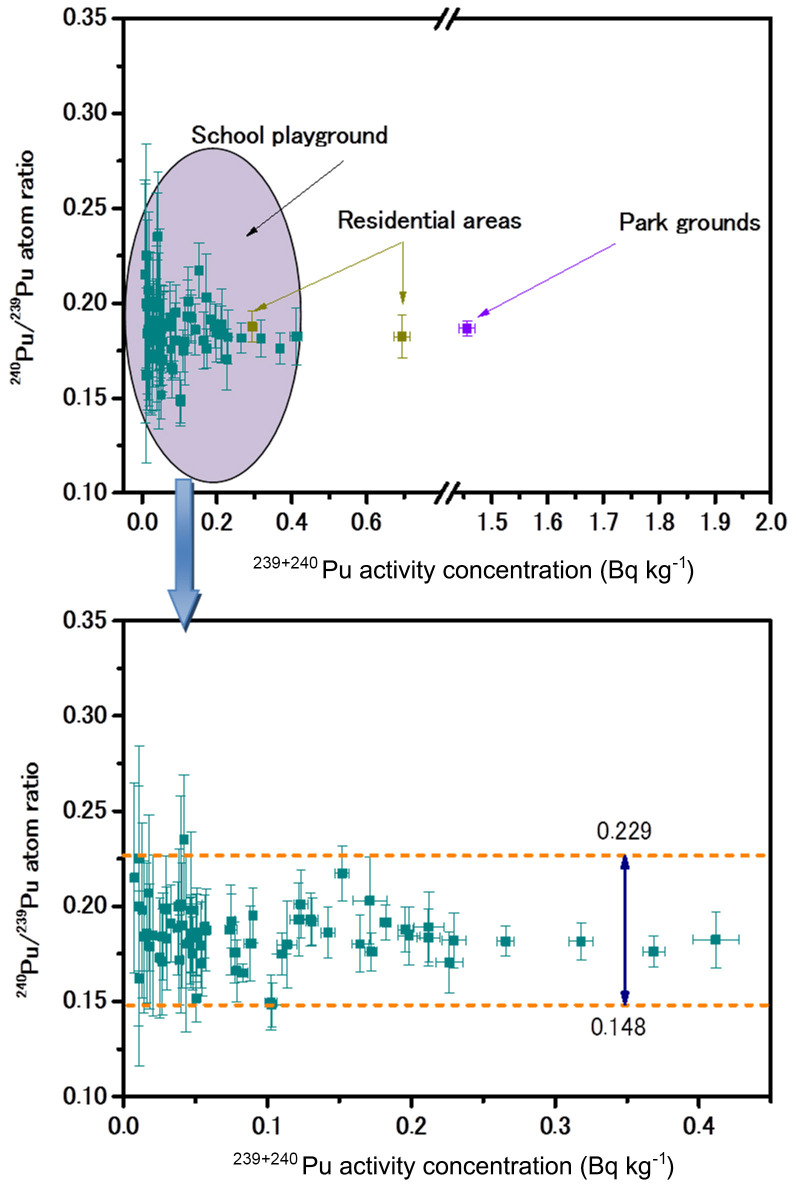
Plot showing the relationship between the ^240^Pu/^239^Pu atom ratio and the ^239+240^Pu activity concentration in soils samples (school grounds, residential areas and park grounds) collected in central-eastern Japan in the 1970s.

**Figure 4:**
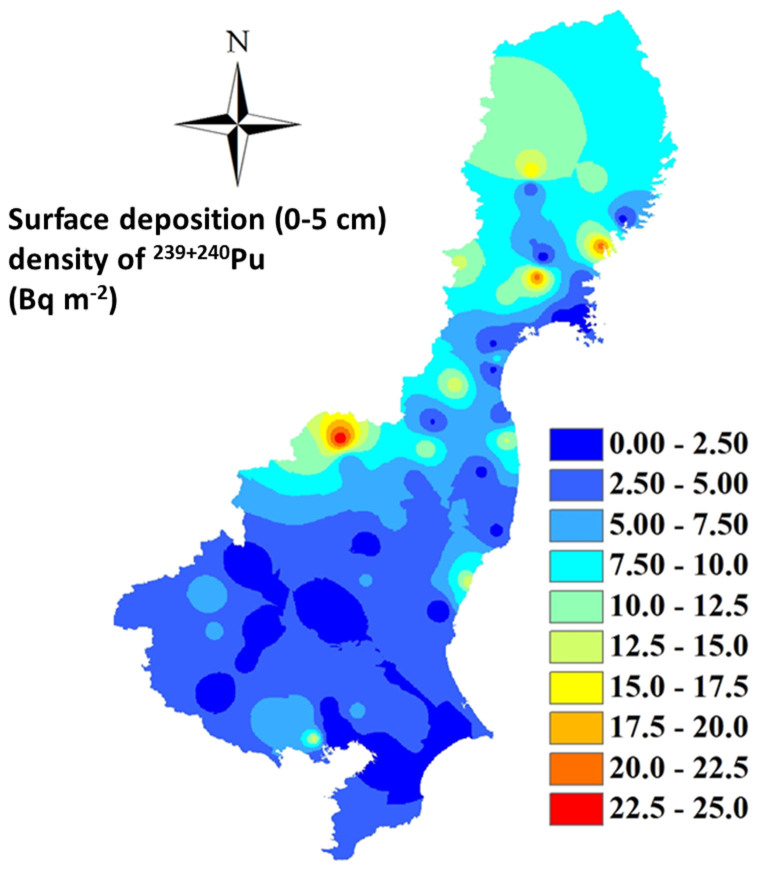
Distribution of the surface (0–5 cm) deposition density of ^239+240^Pu (Bq m^−2^) in soil samples in the central-east Japan in the 1970s. This map was prepared with Arc GIS 10.0 software.

**Figure 5:**
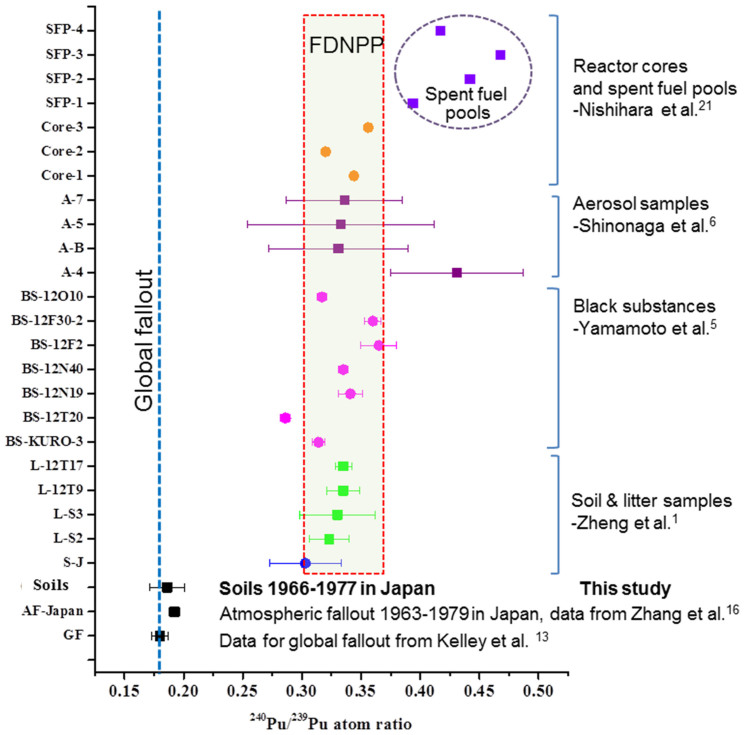
Comparison of the atom ratios of ^240^Pu/^239^Pu in different samples contaminated by the FDNPP accident and the background data of ^240^Pu/^239^Pu atom ratios in environmental samples collected before the FDNPP accident. (Data for global fallout are cited from Kelley et al.^13^; data for atmospheric fallout in Japan (1963–1979) are cited from Zhang et al.^16^; data in surface soil and litter are cited from Zheng et al.^1^ and Yamamoto et al.^5^; data in black substances are cited from Yamamoto et al.^5^; data in aerosol are cited from Shinonaga et al.^6^; and data in reactor cores and spent fuel pools are cited from Nishihara et al.^21^).

**Figure 6:**
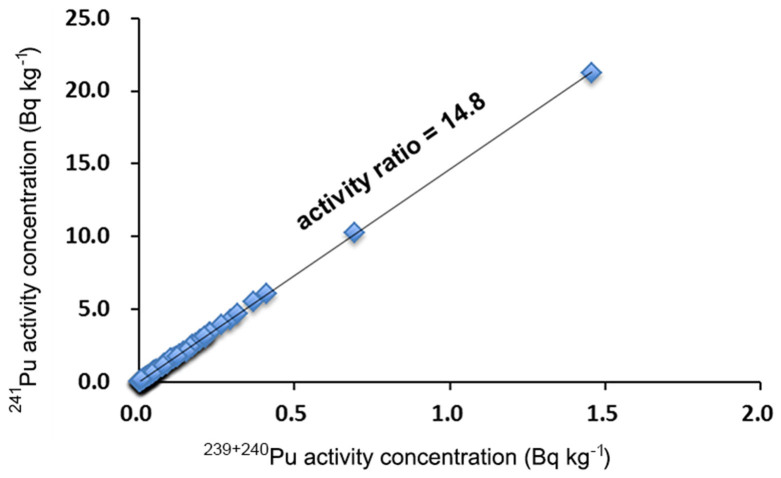
Estimated activity ratios of ^241^Pu/^239+240^Pu in school grounds soil samples collected in central-eastern Japan during the period of peak global fallout in 1964.

**Figure 7:**
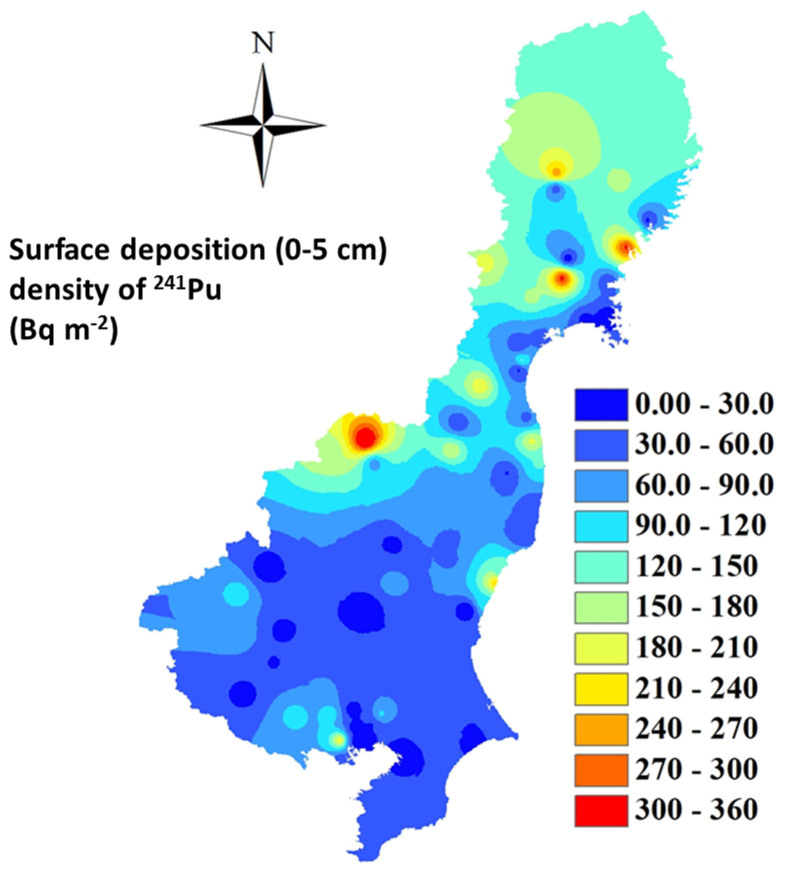
Estimated distribution of the surface (0–5 cm) deposition density of ^241^Pu (Bq m^−2^) in soil samples collected in central-eastern Japan in the period of peak global fallout in 1964. This map was prepared with Arc GIS 10.0 software.

As shown in Fig. 1, eighty soil samples were collected from central-eastern Japan (Fukushima, Ibaraki, Miyagi, Iwata, Tochigi, Gunma, Saitama, and Chiba Prefectures, and Tokyo) in the period from 1969 to 1977. Among them, two were from residential areas, one from a park, and others from school grounds. All samples were analyzed for ^239+240^Pu concentrations and their isotope ratios of ^240^Pu/^239^Pu.

### Instrumentation

A high efficiency sample introduction system (APEX-Q) equipped with a conical concentric nebulizer was combined with SF-ICP-MS (Thermo Fisher Scientific, Element 2, Bremen, Germany) for Pu isotope analysis^24^. This system consisted of a heated cyclonic spray chamber, a Peltier cooled condenser and an ACM Nafion fluoropolymer membrane desolvation module. A small flow of nitrogen was used to increase transport efficiency and signal stability. The low resolution mode was used to utilize the maximal instrument sensitivity. All the measurements were made in the self-aspiration mode with an uptake rate of ~ 0.2 mL min^−1^ to reduce the risk of contamination from the peristaltic pump tubing. The SF-ICP-MS was optimized on a daily basis using 0.1 ng mL^−1^ U standard solution to provide optimum intensities and peak shapes.

### Analytical procedure

The full experimental procedure was described elsewhere^25^. In brief, the soil samples were dried at 105°C for 24 h, and pulverized to about 80 mesh. About 1–3 g of a dried soil sample was weighed out. After ashing at 450°C for 5 h to destroy the organic matter, ca. 1 pg ^242^Pu was added for each sample as a yield monitor. HNO_3_ (20–40 mL, conc.) leaching at 180°C for at least 4 h was done in a tightened lidded Teflon vessel (120 mL, Savillex Corporation, Minnesota, USA) to avoid the loss of acid and improve the acid leaching efficiency. After cooling, the supernatant was filtered through an Advantec filter into a beaker (100 mL), and the Teflon vessel and filter paper were washed with 10–20 mL concentrated HNO_3_. High-purity water was added to adjust the sample solution to the concentration of 8 M HNO_3_. Then, NaNO_2_ was added to a concentration of 0.2 M and heated at 40°C for 30 min to adjust Pu to the tetravalent state prior to loading onto the first AG 1X8 resin column.

The AG 1X8 resin column (2.5 mL) was preconditioned with 20 mL 8 M HNO_3_-0.2 M NaNO_2_. After sample loading, 50 mL 8 M HNO_3_ was used to wash U, Pb and Fe from the column^26^. Then 30 mL 10 M HCl was used for washing Th and converting the resin back into the chloride form. Finally, Pu was eluted with 40 mL 0.1 M NH_4_I-8.5 M HCl, collected in a 100 mL Teflon beaker and evaporated to near dryness. Aqua regia (1 mL) was added and then the solution was heated to dryness again. This procedure was repeated twice to destroy the organic matter and remove the residual iodine. Subsequently, 2 mL concentrated HCl was added and the mixture was evaporated to dryness. After adding ca. 4 mL freshly prepared HCl-H_2_O_2_ solution (10 mL conc. HCl with 0.01 mL 30% H_2_O_2_) and heating at 40°C for about 30 min, the sample solution was ready for loading onto the second AG MP-1M resin column.

The AG MP-1M resin column (2.5 mL) was pre-conditioned with 8 mL HCl-H_2_O_2_ solution. After sample loading, 20 mL 8 M HNO_3_ was used for washing U. Then, 8 mL 10 M HCl was added to wash the residual HNO_3_ in the column and for further Th washing. Pu was eluted from the column with 16 mL HBr into a 30 mL Teflon beaker. After evaporating to near dryness, 1 mL concentrated HNO_3_ was added to the Teflon beaker and heated to remove any trace of HBr. When nearly dry, the final residual was dissolved in 0.8 mL 4% HNO_3_ in preparation for the SF-ICP-MS analysis.

## Author Contributions

J.Z., K.T. and S.U. designed the study. G.S.Y. conducted the Pu analysis. G.S.Y. and J.Z. wrote the manuscript. K.T. and S.U. discussed the results and commented on the manuscript.

## Supplementary Material

Supplementary Information Supplementary Information

## Figures and Tables

**Table 1:** Mean values of Pu activity concentrations and atom ratios in soils of each Prefecture and Tokyo

Prefecture	Sample size	^239+240^Pu activity concentratio (Bq kg^−1^)	^240^Pu/^239^Pu atom ratio	^241^Pu activity concentration (Bq kg^−1^)^a^
Fukushima	22	0.127 ± 0.157	0.183 ± 0.011	1.88 ± 2.31
Ibaraki	5	0.134 ± 0.075	0.182 ± 0.009	1.98 ± 1.15
Miyagi	13	0.207 ± 0.387	0.192 ± 0.011	3.03 ± 5.65
Iwate	9	0.154 ± 0.131	0.190 ± 0.015	2.26 ± 1.93
Tochigi	5	0.038 ± 0.032	0.185 ± 0.011	0.56 ± 0.47
Gunma	4	0.055 ± 0.045	0.166 ± 0.018	0.86 ± 0.71
Saitama	6	0.054 ± 0.045	0.180 ± 0.025	0.82 ± 0.69
Chiba	10	0.048 ± 0.039	0.189 ± 0.009	0.70 ± 0.56
Tokyo	6	0.047 ± 0.082	0.194 ± 0.037	0.69 ± 1.20

^a^Activity of ^241^Pu decay corrected to January 1, 1964.

**Table 2:** Pu activity concentrations and atom ratios in soils collected before and after the FDNPP accident

Sample	Site	Collection date	^239+240^Pu activity (Bq kg^−1^-dry)	^240^Pu/^239^Pu atom ratio	^241^Pu activity (Bq kg^−1^-dry)	^241^Pu/^239^Pu atom ratio	Ref.
**After FDNPP accident**							
Contaminated soil (0–2 cm)	South 20 km from FDNPP	4/20/2011	0.059 ± 0.004	0.303 ± 0.030	4.52 ± 0.56^a^	0.103 ± 0.013^a^	1
Non-contaminated soil	Chiba, Mito, Japan	5/20/2011–8/9/2011	0.016–1.400	0.144–0.209	ND	ND	1
Top soil	Minamisoma, Japan	10/31/2011		0.205 ± 0.039			3
Road dust	Fukushima, Japan	2012	0.145–1.14				4
Road dust	Fukushima, Japan	2012–2013	0.013–3.92	0.286–0.365			5
Soil	Fukushima, Japan	3/27/2011–5/25/2011	0.001–0.353				8
Soil (0–10 cm)	Fukushima, Japan	10/6/2011–6/30/2013	<1.71				5
**Before FDNPP accident**							
Soil	Tokyo, Japan	1970/1971		0.1755 ± 0.0012		0.00171 ± 0.00010^c^	13
Soil	Sapporo, Japan	1970/1971		0.1765 ± 0.0011		0.00183 ± 0.00011^c^	13
Rice-field surface soils^b^	Japan	1957–1980	0.078–1.43				9
Soil	Central-eastern Japan	1969–1977	0.004–1.46	0.186 ± 0.015			This study
Upland fields soils (0–100 cm)	Rokkasho, Japan	2001	0.07–0.7	0.18 ± 0.04			10
Campus surface soil	Kyushu, Japan	1995	0.50–0.65	0.19 ± 0.05			11
Agricultural fields and forests soil	Japan		0.15–4.31	0.155–0.194			12
Atmospheric fallout in Japan		1963–1979		0.1922 ± 0.0044		0.00287 ± 0.00056^c^	16
Global fallout	30–71^0^N			0.180 ± 0.007		0.00194 ± 0.00014^c^	13
	0–30^0^N			0.178 ± 0.010		0.00188 ± 0.00039^c^	13

^a^Decay of^ 241^Pu corrected to March15, 2011.

^b^Soil samples from surface to a depth of 12–20 cm.

^c^Decay of ^241^Pu corrected to January 1, 2000.
