# Supplementary material for: Plutonium concentration and isotopic ratio in soil samples from central-eastern Japan collected around the 1970s
Source: Sci Rep. 2015 Apr 16;5:9636. doi: 10.1038/srep09636 (PMC4399503; doi:10.1038/srep09636)
Supplement: Supplementary Information [file srep09636-s1.doc]

**Supplementary information**

**Plutonium concentration and isotopic ratio in soil samples from central-eastern Japan collected around the 1970s**

Guosheng Yang, Jian Zheng*, Keiko Tagami, Shigeo Uchida

Research Center of Radiological Protection

National Institute of Radiological Sciences,

Anagawa 4-9-1, Inage, Chiba 263-8555, Japan

______________________________________________________________________

*Corresponding author. Tel.: +81 43 2064605; Fax: +81 43 2064601.

Email address: [jzheng@nirs.go.jp](mailto:jzheng@nirs.go.jp)

Table S1 | Pu data in the central-east Japanese surface soil.

| **Soil ID** | **Prefecture** | **City or village** | **Site** | **Sampling date** | **^239+240^Pu activity  (Bq kg^-1^)** | **^240^Pu/^239^Pu  atom ratio** | **^241^Pu activity  (Bq kg^-1^)^a^** |
| --- | --- | --- | --- | --- | --- | --- | --- |
| 182 | Fukushima | Fukushima | School | 12/9/1969 | 0.050±0.003 | 0.183±0.023 | 0.74±0.04 |
| 183 | Fukushima | Fukushima | School | 12/9/1969 | 0.022±0.004 | ND | 0.31±0.03 |
| 184 | Fukushima | Aizuwaka-  matsu | School | 10/20/1970 | 0.079±0.003 | 0.166±0.016 | 1.20±0.06 |
| 185 | Fukushima | Koriyama | School | 12/10/1969 | 0.048±0.002 | 0.175±0.019 | 0.72±0.03 |
| 186 | Fukushima | Koriyama | School | 12/10/1969 | 0.038±0.002 | 0.189±0.024 | 0.55±0.03 |
| 187 | Fukushima | Koriyama | School | 12/10/1969 | 0.110±0.003 | 0.175±0.011 | 1.65±0.05 |
| 189 | Fukushima | Iwaki | School | 12/8/1969 | 0.057±0.002 | 0.189±0.016 | 0.82±0.04 |
| 191 | Fukushima | Iwaki | School | 12/8/1969 | 0.033±0.002 | 0.191±0.020 | 0.48±0.03 |
| 192 | Fukushima | Iwaki | School | 12/8/1969 | 0.006±0.001 | ND | 0.09±0.01 |
| 193 | Fukushima | Shirakawa | School | 12/10/1969 | 0.090±0.003 | 0.195±0.014 | 1.29±0.03 |
| 194 | Fukushima | Haramachi | School | 12/9/1969 | 0.196±0.005 | 0.188±0.012 | 2.85±0.07 |
| 196 | Fukushima | Kitakata | School | 10/20/1970 | 0.368±0.008 | 0.176±0.008 | 5.50±0.12 |
| 197 | Fukushima | Soma | School | 12/9/1969 | 0.054±0.003 | 0.180±0.023 | 0.80±0.05 |
| 198 | Fukushima | Nihonmatsu | School | 12/10/1969 | 0.182±0.004 | 0.192±0.009 | 2.63±0.06 |
| 199 | Fukushima | Nishiaizu | School | 10/20/1970 | 0.172±0.004 | 0.176±0.010 | 2.57±0.05 |
| 202 | Fukushima | Yamatsuri | School | 12/10/1969 | 0.058±0.003 | 0.187±0.022 | 0.84±0.03 |
| 203 | Fukushima | Miyakoji | School | 12/9/1969 | 0.030±0.002 | 0.183±0.027 | 0.45±0.04 |
| 204 | Fukushima | Hirono | School | 12/8/1969 | 0.039±0.003 | 0.200±0.030 | 0.56±0.05 |
| 205 | Fukushima | Okuma | Residential area | 12/8/1969 | 0.695±0.022 | 0.182±0.011 | 10.24±0.44 |
| 206 | Fukushima | Futaba | Residential area | 12/9/1969 | 0.294±0.007 | 0.188±0.008 | 4.28±0.13 |
| 207 | Fukushima | Namie | School | 12/9/1969 | 0.131±0.004 | 0.192±0.012 | 1.89±0.07 |
| 208 | Fukushima | Odaka | School | 12/9/1969 | 0.051±0.002 | 0.152±0.012 | 0.80±0.04 |
| 210 | Ibaraki | Mito | School | 11/30/1976 | 0.044±0.005 | 0.180±0.046 | 0.65±0.10 |
| 211 | Ibaraki | Hitachi | School | 12/10/1969 | 0.122±0.006 | 0.193±0.019 | 1.75±0.11 |
| 223 | Ibaraki | Hitachiota | School | 11/29/1976 | ND | ND |  |
| 224 | Ibaraki | Takahagi | School | 12/8/1969 | 0.227±0.010 | 0.171±0.016 | 3.43±0.16 |
| 225 | Ibaraki | Kitaibaraki | School | 12/8/1969 | 0.142±0.005 | 0.186±0.013 | 2.08±0.10 |
| 130 | Miyagi | Sendai | School | 5/6/1970 | 0.046±0.003 | 0.182±0.023 | 0.68±0.04 |
| 131 | Miyagi | Sendai | School | 5/6/1970 | 0.018±0.001 | 0.179±0.033 | 0.28±0.02 |
| 132 | Miyagi | Sendai | Park ground | 5/6/1970 | 1.456±0.015 | 0.187±0.004 | 21.25±0.25 |
| 133 | Miyagi | Ishinomaki | School | 5/7/1970 | 0.021±0.002 | ND | 0.32±0.06 |
| 134 | Miyagi | Shiogama | School | 5/7/1970 | 0.047±0.003 | 0.186±0.020 | 0.69±0.05 |
| 135 | Miyagi | Furukawa | School | 5/17/1970 | 0.171±0.012 | 0.203±0.023 | 2.45±0.22 |
| 136 | Miyagi | Kesennuma | School | 5/8/1970 | 0.318±0.008 | 0.182±0.010 | 4.69±0.154 |
| 137 | Miyagi | Shiroishi | School | 12/9/1969 | 0.212±0.010 | 0.189±0.019 | 3.08±0.19 |
| 138 | Miyagi | Natori | School | 5/17/1970 | 0.130±0.005 | 0.193±0.014 | 1.88±0.08 |
| 139 | Miyagi | Iwanuma | School | 12/9/1969 | 0.029±0.002 | 0.198±0.028 | 0.42±0.032 |
| 140 | Miyagi | Naruko | School | 5/17/1970 | 0.198±0.008 | 0.184±0.015 | 2.91±0.14 |
| 141 | Miyagi | Onagawa | School | 5/7/1970 | 0.008±0.001 | 0.215±0.050 | 0.11±0.01 |
| 143 | Miyagi | Shizugawa | School | 5/7/1970 | 0.040±0.015 | 0.201±0.057 | 0.60±0.36 |
| 110 | Iwate | Morioka | School | 5/9/1970 | 0.230±0.008 | 0.182±0.014 | 3.39±0.13 |
| 111 | Iwate | Morioka | School | 5/9/1970 | 0.412±0.016 | 0.182±0.015 | 6.07±0.30 |
| 114 | Iwate | Ofunato | School | 5/8/1970 | 0.027±0.002 | 0.171±0.028 | 0.41±0.03 |
| 115 | Iwate | Mizusawa | School | 5/16/1970 | 0.018±0.003 | 0.207±0.041 | 0.26±0.05 |
| 116 | Iwate | Hanamaki | School | 5/16/1970 | 0.266±0.006 | 0.182±0.008 | 3.92±0.12 |
| 117 | Iwate | Kitakami | School | 5/16/1970 | 0.047±0.005 | 0.198±0.041 | 0.69±0.05 |
| 120 | Iwate | Tono | School | 5/8/1970 | 0.164±0.006 | 0.180±0.015 | 2.43±0.07 |
| 121 | Iwate | Ichinoseki | School | 5/16/1970 | 0.075±0.002 | 0.192±0.014 | 1.08±0.03 |
| 122 | Iwate | Rikuzen-  takata | School | 5/8/1970 | 0.152±0.005 | 0.217±0.014 | 2.08±0.09 |
| 231 | Tochigi | Utsunomiya | School | 10/18/1976 | 0.007±0.001 | ND | 0.11±0.02 |
| 241 | Tochigi | Otawara | School | 10/19/1976 | 0.041±0.003 | 0.190±0.033 | 0.61±0.06 |
| 242 | Tochigi | Yaita | School | 10/19/1976 | 0.039±0.003 | 0.172±0.024 | 0.59±0.05 |
| 243 | Tochigi | Tochigi | School | 10/19/1976 | 0.088±0.004 | 0.180±0.020 | 1.31±0.06 |
| 244 | Tochigi | Nasushiobara | School | 10/19/1976 | 0.013±0.002 | 0.198±0.046 | 0.20±0.03 |
| 246 | Gunma | Maebashi | School | 12/1/1976 | 0.083±0.004 | 0.165±0.005 | 1.27±0.05 |
| 249 | Gunma | Kiryu | School | 11/30/1976 | 0.021±0.002 | 0.185±0.042 | 0.30±0.03 |
| 252 | Gunma | Numata | School | 10/20/1976 | 0.103±0.003 | 0.148±0.011 | 1.64±0.04 |
| 260 | Gunma | Katashina | School | 10/20/1976 | 0.014±0.002 | ND | 0.22±0.03 |
| 262 | Saitama | Kumagaya | School | 12/1/1976 | 0.055±0.002 | 0.170±0.017 | 0.83±0.04 |
| 265 | Saitama | Urawa | School | 3/14/1977 | 0.114±0.007 | 0.180±0.023 | 1.69±0.13 |
| 268 | Saitama | Chichibu | School | 3/16/1977 | 0.016±0.002 | 0.186±0.037 | 0.25±0.03 |
| 276 | Saitama | Sayama | School | 12/2/1976 | 0.103±0.004 | 0.149±0.014 | 1.63±0.06 |
| 278 | Saitama | Fukaya | School | 12/1/1976 | 0.025±0.002 | 0.173±0.032 | 0.38±0.04 |
| 281 | Saitama | Koshigaya | School | 8/31/1973 | 0.011±0.002 | 0.225±0.059 | 0.16±0.02 |
| 290 | Chiba | Chiba | School | 8/27/1973 | 0.008±0.002 | ND | 0.12±0.02 |
| 291 | Chiba | Chiba | School | 8/27/1973 | ND | ND |  |
| 292 | Chiba | Choshi | School | 8/29/1973 | 0.075±0.004 | 0.188±0.024 | 1.09±0.05 |
| 293 | Chiba | Choshi | School | 8/29/1973 | 0.010±0.002 | ND | 0.15±0.02 |
| 303 | Chiba | Matsudo | School | 8/31/1973 | 0.010±0.001 | 0.200±0.063 | 0.15±0.02 |
| 304 | Chiba | Matsudo | School | 8/31/1973 | 0.047±0.002 | 0.185±0.020 | 0.69±0.03 |
| 308 | Chiba | Narita | School | 8/30/1973 | 0.051±0.003 | 0.186±0.023 | 0.75±0.04 |
| 311 | Chiba | Yokaichiba | School | 8/29/1973 | 0.027±0.002 | 0.184±0.023 | 0.41±0.03 |
| 315 | Chiba | Kashiwa | School | 8/31/1973 | 0.123±0.005 | 0.201±0.018 | 1.75±0.08 |
| 316 | Chiba | Kashiwa | School | 8/31/1973 | 0.077±0.003 | 0.176±0.016 | 1.16±0.07 |
| 334 | Tokyo | Shinjuku | School | 3/18/1977 | 0.212±0.008 | 0.184±0.015 | 3.12±0.11 |
| 338 | Tokyo | Koto | School | 3/30/1977 | 0.004±0.001 | ND | 0.06±0.01 |
| 339 | Tokyo | Koto | School | 3/30/1977 | 0.006±0.001 | ND | 0.10±0.02 |
| 343 | Tokyo | Ota | School | 2/24/1977 | 0.042±0.003 | 0.229±0.029 | 0.56±0.04 |
| 358 | Tokyo | Adachi | School | 3/14/1977 | 0.011±0.001 | 0.162±0.046 | 0.18±0.02 |
| 362 | Tokyo | Edogawa | School | 3/30/1977 | 0.009±0.001 | ND | 0.15±0.01 |

^a^ ^241^Pu decay corrected to January 1, 1964.

^241^Pu activities were estimated based on the atom ratio of ^241^Pu/^239^Pu obtained in the fallout deposition samples collected at 14 sites in Japan from 1963 to1979 ^1^.

**References**

1. Zhang, Y. S. et al. Characterization of Pu concentration and its isotopic composition in a reference fallout material. *Sci. Total Environ.* 408, 1139-1144 (2010).
